# Thrombocytopenia induced by glycoprotein (GP) IIb-IIIa antagonists: about two cases

**DOI:** 10.11604/pamj.2021.38.9.27215

**Published:** 2021-01-05

**Authors:** Salma Abdeladim, Mahassine Elharras, Amal Elouarradi, Ilham Bensahi, Sara Oualim, Fatimazahra Merzouk, Mohamed Sabry

**Affiliations:** 1 Department of Cardiology, Cheick Khalifa International University Hospital, Mohammed VI University of Health Sciences (UM6SS), Casablanca, Morocco

**Keywords:** Glycoprotein IIb/IIIa receptor antagonist, thrombocytopenia, tirofiban, case report

## Abstract

In this paper, we report two cases of induced thrombocytopenia after the infusion of glycoprotein (GP) IIb/IIIa receptors antagonists, following a coronary angioplasty. The first patient is a 65-year-old woman, admitted with acute coronary syndrome requiring percutaneous angioplasty with stenting. The patient was given tirofiban + unfractionated heparin (UFH). Ten hours later, the patient revealed very severe thrombocytopenia and went into hemorrhagic shock (hematemesis and hematoma at the injection site). The patient was transfused with nine units of red blood cells (RBCs), 24 platelets pellets and 4 units of fresh frozen plasma (FFP). The second patient is a 76-year-old woman. She was admitted to hospital for acute coronary syndrome necessitating percutaneous angioplasty with stenting and a glycoprotein IIb/IIIa receptor antagonists, tirofiban + unfractionated (UFH). Four hours later, the patient presented with gingivorrhagia associated thrombocytopenia. She received six platelet pellets transfusion with well clinical and biological improvement. These two observations raise the significance of a close monitoring of platelet count after the initiation of GP IIb/IIIa antagonists infusion, which are sometimes responsible for life-threatening adverse events.

## Introduction

Glycoprotein IIb/IIIa receptor antagonists are platelet anti-aggregant, which are nowadays increasingly being used in the treatment of acute coronary syndrome (ACS) and after a percutaneous coronary intervention (PCI) [1]. Thrombocytopenia is a common complication but rare within this therapeutic class [2]. We report two cases of thrombocytopenia with different severity degrees after tirofiban treatment in two patients with ACS undergoing a percutaneous coronary intervention (PCI).

## Patient and observation

**Case 1:** a 65-year-old patient on beta-blocker for hypertension as a major cardiovascular risk factor. She was initially admitted for non-ST elevation myocardial infarction (NSTEMI) with negative troponin associated with a tight stenosis of the moderate LAD at the onset of the diagonal. She underwent coronary angioplasty with stenting. At the end of the procedure, the patient presented with chest pain with an upward shift of the ST segment and hemodynamic instability. Angiographically, it was an extensive stent thrombosis and an upstream stent requiring balloon permeabilization. Then, a decision was made to put the patient on tirofiban and unfractionated heparin. Tirofiban was intravenously administrated at a dose of 0.4 μg/kg/min for 30 min followed by 0.1 μg/kg/min continuous infusion.

**Figure 1:**
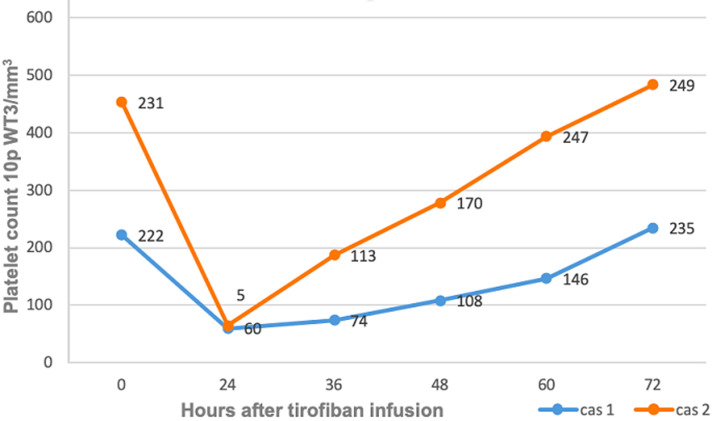
platelet count changes after tirofiban infusion

On hospital admission, the patient had a normal complete blood count (CBC), including platelet count (228 × 10^3^/mm^3^ [n=(150-400) × 10^3^/mm^3^]). Her renal function was normal. Ten hours after the catheterization, the patient presented with hemorrhagic shock (hematemesis and hematoma at the injection site) with thrombocytopenia (platelet count 60 × 10^3^/mm^3^), Figure 1 shows the time course of platelet recovery. Accordingly, tirofiban, heparin, clopidogrel and aspirin were discontinued and she was transfused with nine units of red blood cells, 24 platelet pellets and four units of fresh frozen Plasma (FFP). Three days later, hemodynamic status stabilized, laboratory test was back to normal and it was decided to resume the anticoagulation therapy + dual antiplatelet aggregation and to stop aspirin after 4 weeks.

**Case 2**: 76-year-old patient, hypertensive, with no other significant past medical history. She was admitted for NSTEMI with troponin positive associated with an acute occlusion of the right coronary artery. The patient underwent angioplasty with stenting. Since the clot burden was large, it was decided to administer tirofiban and unfractionated heparin. Four hours later, the patient presented with gingivorrhagia with severe thrombocytopenia on laboratory tests (platelet count 5 × 10^3^/mm^3^). Tirofiban and UFH were stopped and the patient received a transfusion of six platelet pellets. After 12 hours of stopping tirofiban, the platelet count had increased to 113 × 10^3^/mm^3^ /l (Figure 1). There was no fall in hemoglobin, no recurrence of hemorrhage. The hospital course of the patient was uneventful and she was discharged home with normal hematological test results (platelet count, 247 × 10^3^/mm^3^). Her one-week blood test revealed microcytic hypochromic anemia at 8g/dl requiring the transfusion of two units of red blood cells. The platelet count was correct while taking aspirin and clopidogrel.

## Discussion

Glycoprotein (GP) IIb/IIIa is the most abundant receptor expressed on platelet and megakaryocyte membranes. Therefore, inhibition of GP IIb/IIIa is described as a very effective approach in antiplatelet therapy [3]. Glycoprotein IIb/IIIa inhibitors are widely used in the treatment of patients with ACS and during percutaneous coronary intervention (PCI) procedures. Tirofiban which is the most used glycoprotein IIb/IIIa inhibitors in our country, is a non-peptide molecule, which reversibly inhibits platelet aggregation by binding to GP IIb/IIIa receptors. By blocking the glycoprotein IIb/IIIa receptor, tirofiban blocks the final essential step for platelet aggregation, particularly, the binding of plasmatic fibrinogen or Von Willebrand factor binding to this activated membrane protein. Thus, the fibrinogen molecule prevented the platelets crosslinking [4]. Thereby, decreasing ischemic complications and mortality associated with ACS and PCI. On the other hand, adverse reactions to these agents have been identified, namely bleeding and thrombocytopenia. Acute thrombocytopenia is a common side effect of the three clinically approved inhibitors: tirofiban, eptifibatide and abciximab. Five patterns of GPII/bIIIa inhibitors-induced thrombocytopenia have been identified [5,6].

The pathogenesis of this platelet destruction, in most cases, can be secondary to the development of circulating antibodies against IIb/IIIa antagonists [5,7-10]. These antibodies react with IIb/IIIa antagonist-coated platelets and cause their destruction. Thrombocytopenia occurring after first exposure to a GPIIb/IIIa inhibitor seems to be explained by the fact that antibodies are naturally present in some normal individuals. Delayed onset of thrombocytopenia is explained by persistence of platelet-bound drug for several weeks after treatment, rendering platelets susceptible to be destructed by newly formed antibodies [9]. The incidence of thrombocytopenia, defined as an absolute platelet count of <90 × 10^9^/l, was 1.1% in the PRISM study [11], 1.9% in PRISM-PLUS study [12] and 1.1% in RESTORE study [13]. Severe thrombocytopenia (platelet count, <50 × 10^9^/l) has occurred in 0.5% of patients in clinical trials.

Multiple tests have been used to show that most patients developing thrombocytopenia after treatment with a GP IIb/IIIa inhibitor have antibodies that recognize GP IIb/IIIa occupied by the provocative drug. The favored method involves flow cytometric detection of immunoglobulins in patient´s serum that bind to normal platelets in the presence of the implicated drug. It is important to monitor platelet counts closely after initiation of anti-GP IIb/IIIa. Monitoring of platelet counts at 6, 12 and 24 hours will detect most cases of acute thrombocytopenia. Our two patients developed thrombocytopenia a few hours after administration of tirofiban + UFH (after 10 hours for the first case and after four hours for the second). The possibility of heparin-induced thrombocytopenia (HIT) was low. HIT generally results from the formation of antiplatelet antibodies and thrombocytopenia generally develops after 5 days in naive patients and within minutes to hours of exposure in those who have received heparin therapy within the past 6 months. No one of our patients has been exposed to any heparins previously.

Isolated profound thrombocytopenia is rarely known with aspirin and clopidogrel [14]. The mechanism in these cases is often due to induction of some sensitizing antibodies at the time of preceding treatment with these drugs. Yet, our two patients had received only one dose of aspirin and clopidogrel and risk of developing sensitizing antibodies is very low. Moreover, this dual antiplatelet aggregation was continued in the two patients after the correction of their hematological tests without any recurrence of thrombocytopenia. Additionally, the fact that platelet count in our patient rapidly increased after stopping tirofiban infusion confirms the hypothesis of immune-mediated thrombocytopenia. As soon as thrombocytopenia is detected, it is obligatory to stop tirofiban infusion. Upon cessation of treatment, platelet levels return to normal in two to five days [15]. The therapeutic management of thrombocytopenia often makes use of platelet transfusions as recommended by the European Society of Cardiology [1]. In case of moderate thrombocytopenia, simple monitoring with cessation of other therapies modifying hemostasis may be considered [2]. Platelet pellets transfusion is recommended when the platelet count is below 50 G/L and active bleeding [1]. In rare cases, corticosteroid or normal human immunoglobulins therapies have been successfully used associated with a short time course of thrombocytopenia.

## Conclusion

Glycoprotein IIb/IIIa receptor antagonists (abciximab, eptifibatide, tirofiban) are platelet anti-aggregants that have been proved effective in decreasing ischemic complications of percutaneous coronary interventions, but they are also associated with a risk of sudden onset of thrombocytopenia, hence the need of close monitoring after their infusion. The mechanism of this thrombocytopenia is still less understood. Further studies are necessary to answer some unresolved questions and improve antibody detection.

## References

[1] Roffi M, Patrono C, Collet J-P, Mueller C, Valgimigli M, Andreotti F (2016). 2015, ESC Guidelines for the management of acute coronary syndromes in patients presenting without persistent ST-segment elevation: task force for the management of acute coronary syndromes in patients presenting without persistent ST-segment elevation of the European Society of Cardiology (ESC). Eur Heart J.

[2] Huxtable LM, Tafreshi MJ, Rakkar ANS (2006). Frequency and management of thrombocytopenia with the glycoprotein IIb/IIIa receptor antagonists. Am J Cardiol.

[3] Xiang Q, Pang X, Liu Z, Yang G, Tao W, Pei Q (2019). Progress in the development of antiplatelet agents: Focus on the targeted molecular pathway from bench to clinic. Pharmacol Ther.

[4] Lynch JJ, Cook JJ, Sitko GR, Holahan MA, Ramjit DR, Mellott MJ (1995). Nonpeptide glycoprotein IIb/IIIa inhibitors: antithrombotic effects of MK-0383. J Pharmacol Exp Ther.

[5] Billheimer JT, Dicker IB, Wynn R, Bradley JD, Cromley DA, Godonis HE (2002). Evidence that thrombocytopenia observed in humans treated with orally bioavailable glycoprotein IIb/IIIa antag-onists is immune mediated. Blood.

[6] Sane DC, Damaraju LV, Topol EJ, Cabot CF, Mascelli MA, Harr-ington RA (2000). Occurrence and clinical significance of pseudothrombocytopenia during abciximab therapy. J Am Coll Cardiol.

[7] Bougie DW, Wilker PR, Wuitschick ED, Curtis BR, Malik M, Levine S (2002). Acute thrombocytopenia after treatment with tirofiban or eptifibatide is associated with antibodies specific for ligand-occupied GPIIb/IIIa. Blood.

[8] Curtis BR, Swyers J, Divgi A, McFarland JG, Aster RH (2002). Thrombocytopenia after second exposure to abciximab is caused by antibodies that recognize abciximab-coated platelets. Blood.

[9] Curtis BR, Divgi A, Garritty M, Aster RH (2004). Delayed thrombo-cytopenia after treatment with abciximab: a distinct clinical entity associated with the immune response to the drug. J Thromb Haemost.

[10] Wang S, Sawalha K, Khan A (2020). An unusual case of drug-induced thrombocytopenia. J Investig Med High Impact Case Rep.

[11] Platelet Receptor Inhibition in Ischemic Syndrome Management (PRISM) Study Investigators (1998). A comparison of aspirin plus tirofiban with aspirin plus heparin for unstable angina. N Engl J Med.

[12] Platelet receptor inhibition in ischemic syndrome management in patients limited by unstable signs and symptoms (PRISM-PLUS) study investigators (1998). Inhibition of the platelet glycoprotein IIb/IIIa receptor with tirofiban in unstable angina and non-Q-wave myocardial infarction. N Engl J Med.

[13] The RESTORE investigators: randomized efficacy study of tirofiban for outcomes and restenosis (1997). Effects of platelet glycoprotein IIb/IIIa blockade with tirofiban on adverse cardiac events in patients with unstable angina or acute myocardial infarction undergoing coronary angioplasty. Circulation.

[14] Guo YL, Li JJ, Yuan JQ, Qin XW, Zheng X, Mu CW (2010). Profound thrombocytopenia induced by clopidogrel with a prior history of long-term safe administration. World J Cardiol.

[15] Aster RH, Curtis BR, Bougie DW, Dunkley S, Greinacher A, Warkentin TE (2006). Thrombocytopenia associated with the use of GPIIb/IIIa inhibitors: Position paper of the ISTH working group on thrombocytopenia and GPIIb/IIIa inhibitors. J Thromb Haemost.

